# An analysis of real-life data of infants born to mothers with autoimmune thyroiditis: do they need to be followed-up?

**DOI:** 10.1186/s13052-025-01915-x

**Published:** 2025-03-24

**Authors:** Beatrice Righi, Nives Melli, Alessandra Cassio, Alessio Canovi, Francesco Leo, Chiara Sartori, Alessandra Polese, Rossana Colla, Alessandro De Fanti, Giancarlo Gargano, Maria Elisabeth Street

**Affiliations:** 1 Department of Mother and Child, Azienda USL-IRCCS di Reggio Emilia, Reggio Emilia, Italy; 2 Department of Mother and Child, Neonatal Intensive Care Unit, Azienda USL-IRCCS di Reggio Emilia, Reggio Emilia, Italy; 3 Department of Medical and Surgery Sciences, University of Bologna, 40138 Bologna, Italy; 4 Clinical Chemistry and Endocrinology Laboratory, Department of Diagnostic Imaging and Laboratory Medicine, Azienda USL-IRCCS di Reggio Emilia, Via Amendola 2, 42122 Reggio Emilia,, Italy; 5 Department of Medicine and Surgery, University of Parma, Parma, Italy; 6 Paediatrics, University Hospital of Parma, Via Gramsci 14, 43126 Parma, Italy

**Keywords:** Autoimmune thyroiditis, Pregnancy, Maternal hypothyroidism, Neonatal screening

## Abstract

**Background:**

Infants born to mothers with autoimmune thyroiditis (AT) could be at risk of developing thyroid dysfunction, and maternal anti-thyroid antibodies have been shown to have a clinical impact on offspring. We aimed at evaluating the usefulness of our follow-up intervention protocol in newborns from mothers with AT and to define the most appropriate management for these neonates.

**Methods:**

89 mothers with AT and their newborns were included. Data on maternal thyroid function and autoimmunity were collected; serum thyroid function and autoimmunity of infants were assessed regularly until normalisation of thyroid stimulating hormone (TSH) and anti-thyroid antibodies, according to the local protocol.

**Results:**

Thyroid auto-antibodies were measured in 38% and in 62% of mothers before and during pregnancy, respectively. Anti-thyroid peroxidase (anti-TPO) and anti-thyroglobulin (anti-TG) were positive in 97% and 61%, respectively, of the mothers assessed. Anti-TSH receptor antibodies (TRAb) were checked in 18% of the mothers and all were negative. 94% of newborns at first evaluation had positive anti-thyroid antibodies, starting to normalise or decrease from the second month of life. Analysing TSH levels according to the days of postnatal life of collection samples (T1: 30 ± 7 days, T2: 61 ± 9 days, T3: 105 ± 49 days, T4: 135 ± 31 days, T5: 247 ± 64 days), peak TSH levels were found at T4 (4.4 ± 2.2 mU/L), within the cut-off of 6 mU/L. 84% of children maintained a normal thyroid function during follow-up; 12% of infants presented a TSH above 6 mU/L at least in one blood test, showing normalisation during follow-up. Only one infant received replacement therapy for hypothyroidism at 2 months. 91% of the 22 thyroid ultrasounds (US) performed were normal. In those with changes thyroid function normalised anyway.

**Conclusions:**

Mothers with AT do not seem to deliver newborns at risk of overt hypothyroidism. However, because of the possible negative effects of maternal anti-thyroid antibodies, we underline the importance of monitoring thyroid autoimmunity during pregnancy, including both anti-TG besides anti-TPO antibodies.

## Background

During pregnancy, the maternal thyroid gland undergoes modifications: both the size of the thyroid gland and iodine requirement increase, and the synthesis of thyroid hormones is increased. This underlines the importance of normal thyroid function throughout pregnancy [1]. Thyroid antibodies, when present, increase the risk of thyroid dysfunction in the mothers [1]. The detection rate is reported to be 8% in the general female population seeking fertility, 2–17% in pregnant women worldwide and 30 to 60% in pregnant women with a high thyroid stimulating hormone (TSH) [1, 2]. Specifically, anti-thyroid peroxidase (anti-TPO) antibody positivity has been associated with an increased risk of miscarriage, preeclampsia, placental abruption, premature rupture of membranes and preterm birth [1, 3–5]. Other outcomes, such as intrauterine growth restriction (IUGR) and small for gestational age (SGA) newborns, have been described in these mothers but the data in the literature concerning an association between anti-TPO antibodies and birth weight are controversial [6]. Several studies have examined also the association between maternal thyroid autoimmunity and child development [1]. In particular, the offspring of women with anti-TPO positivity has been reported to present lower motor and intellectual development at 25–30 months of life [7], sensorineural hearing loss [8], lower child intelligence quotient (IQ) [9], externalising problems especially attention deficit/hyperactivity problems [10] and autism [11]. Recently, anti-TPO positivity in mothers has also been associated with increased cardiometabolic risk in offspring [12]. Anti-TPO antibodies can be detected in almost all mothers with autoimmune thyroiditis (AT), and are considered to reflect the severity of lymphocyte infiltration in the thyroid gland [2, 13]. Anti-thyroglobulin (anti-TG) antibodies are also detected in AT and have been associated with lower perceptual performance and motor scores in the offspring [1, 14]. Although previous studies have reported an association between anti-TG antibodies and transient congenital hypothyroidism (TCH), suggesting a pathogenetic role of these antibodies [15], their sensitivity and specificity for the diagnosis and prognosis of AT is lower than for anti-TPO [13]. This is the reason why the most recent task force recommendations for the diagnosis and management of thyroid disease during pregnancy and postpartum, suggest to assess only anti-TPO antibodies when testing for thyroid autoimmunity [1]. Anti-TSH receptor antibodies (TRAb) can also be present in mothers with thyroid dysfunction causing maternal hyperthyroidism or, in rare cases, hypothyroidism [16], depending on a stimulating or blocking action of these antibodies on the TSH receptor [2]. Many patients with autoimmune hyperthyroidism have circulating anti-TPO, reflecting concomitant AT [2].

Universal screening for thyroid disease in pregnancy is not recommended [1] and few studies have analysed the changes in thyroid function in newborns born to mothers with AT. The effects of mild untreated hypothyroidism on growth and neurocognitive outcome in newborns and children are not yet completely clear [17]. The importance of Neonatal Screening (NS) during the first 48–72 h of life in all term newborns to detect congenital hypothyroidism for a timely replacement treatment is well known [18]. However, it is yet unclear whether modifications of thyroid function in newborns from mothers with thyroid autoimmune diseases actually occur, and it is controversial whether further thyroid testing after screening is needed in this population.

This study aimed at evaluating the thyroid function of mothers with AT and their offspring during the first months of life to understand which follow-up protocol should be used, based on the analysis of the data collected at our institution over a 2-year period (2015–2017).

## Methods

### Patients and study design

Data from computerized medical records of 104 mothers and their neonates born in the hospitals of our district between October 2015 and December 2017 were evaluated retrospectively.

Inclusion criteria for the study were: diagnosis of AT in the mothers performed before or at any time during pregnancy based on hormonal data and anti-TPO and/or anti-TG positivity, normal neonatal TSH values at NS (cut-off for recall was 9 mU/L).

Exclusion criteria were: preterm delivery (before 36 weeks of gestational age), peri/neonatal critical illnesses, eclampsia, other thyroid dysfunctions (ex. thyroid nodules, Graves’ disease, iatrogenic hypothyroidism…) or other autoimmune diseases in the mothers.

**Fig. 1:**
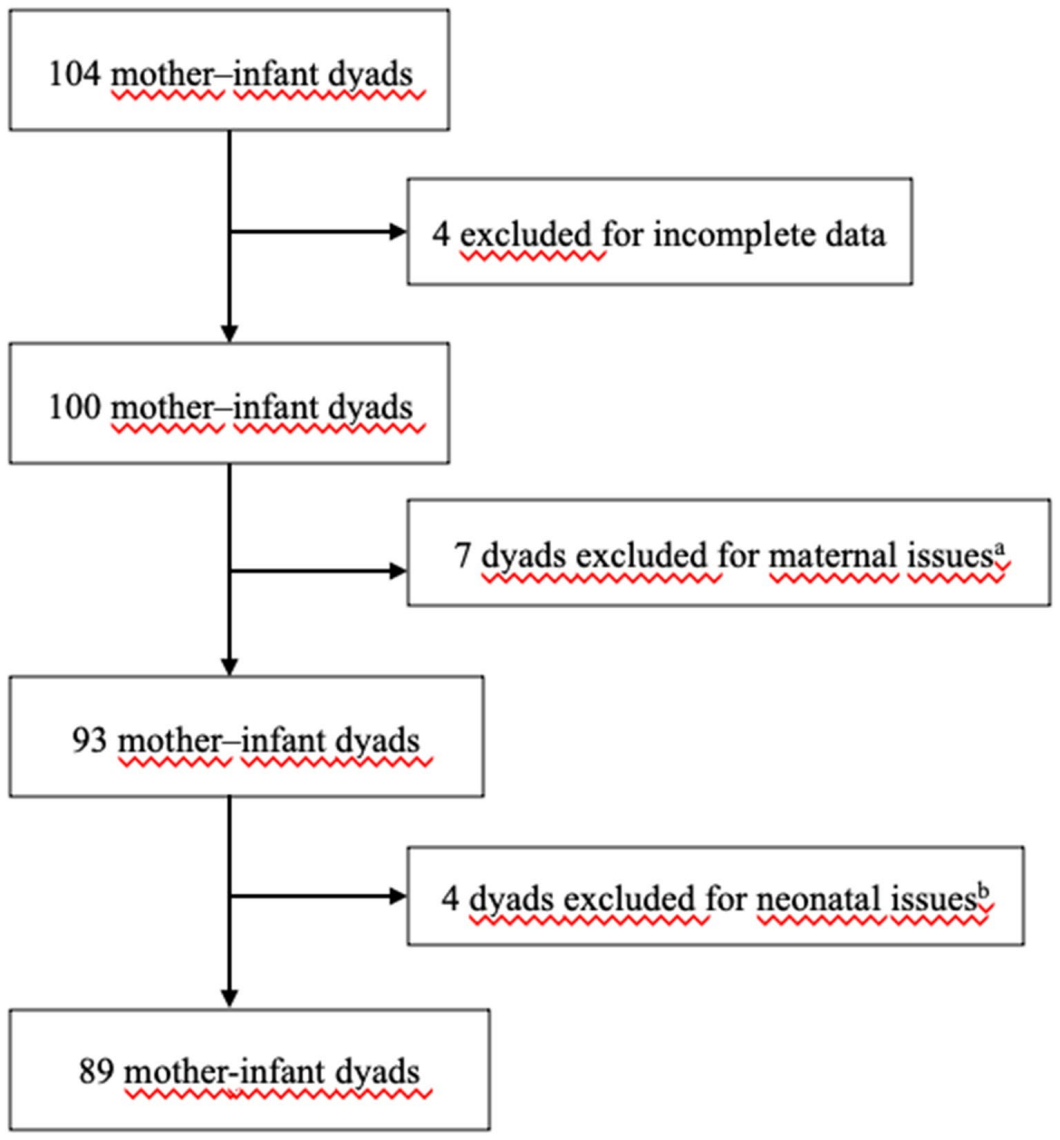
Consort diagram of the study. ^a^ hypothyroidism without positive antibodies (*N* = 6), papillary thyroid carcinoma (*N* = 1). ^b^ prematurity < 36 weeks (*N* = 3), prenatal intraventricular haemorrhage (IVH) (*N* = 1)

Figure 1 shows the flowchart of participant enrollment in the study.

The diagnosis of maternal AT was performed by the referring physician or endocrinologist and the mothers were managed by their gynaecologist/obstetrician or endocrinologist. In the mothers, thyroid function was regularly assessed during pregnancy and treatment with L-thyroxine was started when needed according to the official guidelines and adjusted to maintain hormonal levels within the normal range [1].The following data were also collected in the mothers: the most recent assessment of TSH, free thyroxine (FT4) and free triiodothyronine (FT3), the most recent thyroid antibody titres (anti-TPO, anti-TG and TRAb) assayed at any time before or during pregnancy, use of L-thyroxine during pregnancy.

After the first 7 days of life and within the second month of life, TSH, FT4, FT3 levels, anti-TPO, anti-TG and TRAb were measured at least once in the serum of children according to the local protocol. If antibodies were positive, independent of normal TSH values, subsequent samples for thyroid function and anti-thyroid antibodies were assessed monthly until antibody negativity. The cut-off for serum TSH in neonates and infants at this time was set at 6 mU/L [18]. Anti-TPO and anti-TG antibodies were considered positive if above the upper limit according to local reference levels. Whenever antibodies were positive and/or TSH was above 6 mU/L after the second serum sample, infants were referred to the paediatric endocrinologists.

Thyroid ultrasound (US) was performed at the local hospital only in the newborns with a TSH persistently above 6 mU/L (*N* = 22/89).

In newborns the following information were also recorded: sex, anthropometric parameters (length, weight and head circumference) at birth, mode of delivery (spontaneous delivery, operative delivery or caesarean section), any admission to the neonatal intensive care unit (NICU), and any other neonatal diseases. Neonatal feeding (breastfeeding, bottle feeding or mixed) at birth was also registered.

We identified 5 time-frames (T1: 30 ± 7 days, T2: 61 ± 9 days, T3: 105 ± 49 days, T4: 135 ± 31 days, T5: 247 ± 64 days) according to the days of postnatal life when the blood sample for thyroid function and autoimmunity was collected. We analysed the TSH serum levels of the children in each time-frame, in order to verify when the highest TSH levels were registered. As the blood tests were not repeated in the infants who normalized their thyroid function and autoimmunity, the number of subjects over time decreased.

Finally, we analysed the TSH data considering also the current cut-off for recall now set at 6 mU/L for infants above 21 days of life, as recommended by the most recent Consensus Guidelines on Congenital Hypothyroidism [18]. We identified the number of infants having an increased TSH (> 6 mU/L). We also analysed carefully the hormonal data of the newborns/infants who received the longer follow-ups to identify the best time to detect the highest TSH levels in order to identify those truly deserving follow-up for thyroid function.

### Laboratory assay

TSH, FT4 and FT3 levels were assessed on serum using Siemens TSH3-Ultra and FT4 e FT3 immunochemiluminescent methods on analyzer Siemens ADVIA Centaur XP (Siemens Healthineers, Erlangen, Germany). Analytical sensitivity was 0.001 mIU/L for TSH (reference range 0.35–4.5 mIU/L), 1 pg/mL for FT4 (reference range 8–18 pg/mL), 0,2 pg/mL for FT3 (reference range 2,3 e 4,2 pg/mL). Anti-TPO and anti-TG levels were assessed on serum using Abbott (Abbott| Diagnostics Lake Forest, Illinois, USA) anti-TPO and anti-TG immunochemiluminescent methods on analyzer Architect. Analytical sensitivity was 1 UI/mL for both Anti-TPO (cut-off was < 6 UI/mL) and for anti-TG (cut-off was < 4 UI/mL). TRAb levels were assessed on serum using a Radio Immuno Assay marked with Iodium-125 RSR TRAb CT method on analyzer Becton Dickinson (Becton Dickinson, Franklin Lakes, NJ, USA). Analytical sensitivity was 0.3 UI/mL (cut-off was < 1 UI/mL). The analytical performances of the methods were monitored with daily Internal Quality Control and periodic External Quality Assesment.

### Statistical analysis

Statistical analysis was carried out using the SPSS program version 22. Descriptive statistics was used to summarise the study cohort characteristics. Continuous variables with normal distribution were expressed as mean ± standard deviation (SD), continuous variables without normal distribution were expressed as median [minimum-maximum], categorical variables were presented as frequency (percentage).

More detailed analysis were not required for the purpose of this study.

## Results

### Mother-newborn general characteristics

The main features of the newborns included in the study are reported in Table 1 whereas maternal thyroid function tests and thyroid autoimmunity data are reported in Table 2.

**Table 1:** Features of the 89 newborns included in the study

Neonatal data	
Sex (N females/ N males)	50/39
GA (weeks)	39 [36–41]
Weight (g)	3208.0 ± 441.0
Length (cm)	49.9 ± 2.1
Head circumference (cm)	34.0 ± 1.3
APGAR score at 5 min (N)	10 [5–10]
Growth restriction (N SGA/N IUGR/N both SGAand IUGR)	3/1/3
TSH levels at the neonatal screening test (mU/L)	2.72 ± 1.2 [1.31–6.75]
Mode of delivery (N spontaneous/ N caesarean/N operative)	62/23/3
Complications peri/post-partum (N/total)^a^	24/89
Feeding (N breastfeeding/N mixed nutrition/N bottle-feeding)	75/14/0

^a^ Complications included: jaundice (*N* = 13), transient hypoglycaemia (*N* = 3), transient polycythaemia (*N* = 1), transient tachypnoea (*N* = 1), apnoeas and cyanosis (*N* = 1), cardiac abnormalities (intraventricular defects (*N* = 2), PFO (*N* = 2)), facial dysmorphism (*N* = 1), cryptorchidism (*N* = 1), bradycardia (*N* = 1), sepsis (*N* = 1)

*Abbreviations N* number, *GA* gestational age, *TSH* thyroid stimulating hormone, *PFO* patent foramen ovale, *SGA* small for gestational age, *IUGR* intrauterine growth restriction

**Table 2:** Thyroid function data of the 89 mothers at delivery

Maternal data	
Women on L-thyroxine therapy (% of the total)	82%
Anti-TPO antibody positivity (% of the total)	97%
Anti-TG antibody positivity (% of the total)	61%
TRAb positivity (% of the total)	0%
TSH (mU/L)	1.7 ± 0.9
FT4 (pg/ml)	10.3 ± 4.5
FT3 (pg/ml)	4.5 ± 4.4

*Abbreviations N* number, *Anti-TPO* anti-thyroid peroxidase, *anti-TG* anti-thyroglobulin, *TRAb* anti-TSH receptor *antibodies*, *TSH* thyroid stimulating hormone, *FT4* free thyroxine, *FT3* free triiodothyronine

The mean age of the mothers at delivery was 33.3 ± 5.2 years.

Thyroid auto-antibodies were measured in 55/89 (62%) mothers during pregnancy; in 37 of these 55 (67%), antibodies were measured at delivery. Thirty-four/89 mothers (38%) had antibodies assessed before pregnancy.

Fifty-one/89 mothers (57%) had both anti-TPO and anti-TG positivity. Only in 3/89 women (3%) anti-TG antibodies were the only positive auto-antibodies detected.

### Newborn and infant thyroid autoimmunity during follow-up

At the first control (T1), 84/89 infants (94%) had positive anti-TPO and/or anti-TG antibodies.

Eighty/89 infants (90%) had positive anti-TPO, 36/89 (40%) had positive anti-TG, 32/89 (36%) had positive both anti-TPO and anti-TG antibodies. Thirteen/89 infants (15%) had normalised their antibody levels by 95 ± 44 days after birth, 71/89 (80%) presented decreasing titles during the follow-up starting from 69 ± 24 days after birth. None had positive TRAb at any time during the follow-up.

### Newborn and infant thyroid function pattern during follow-up

**Fig. 2:**
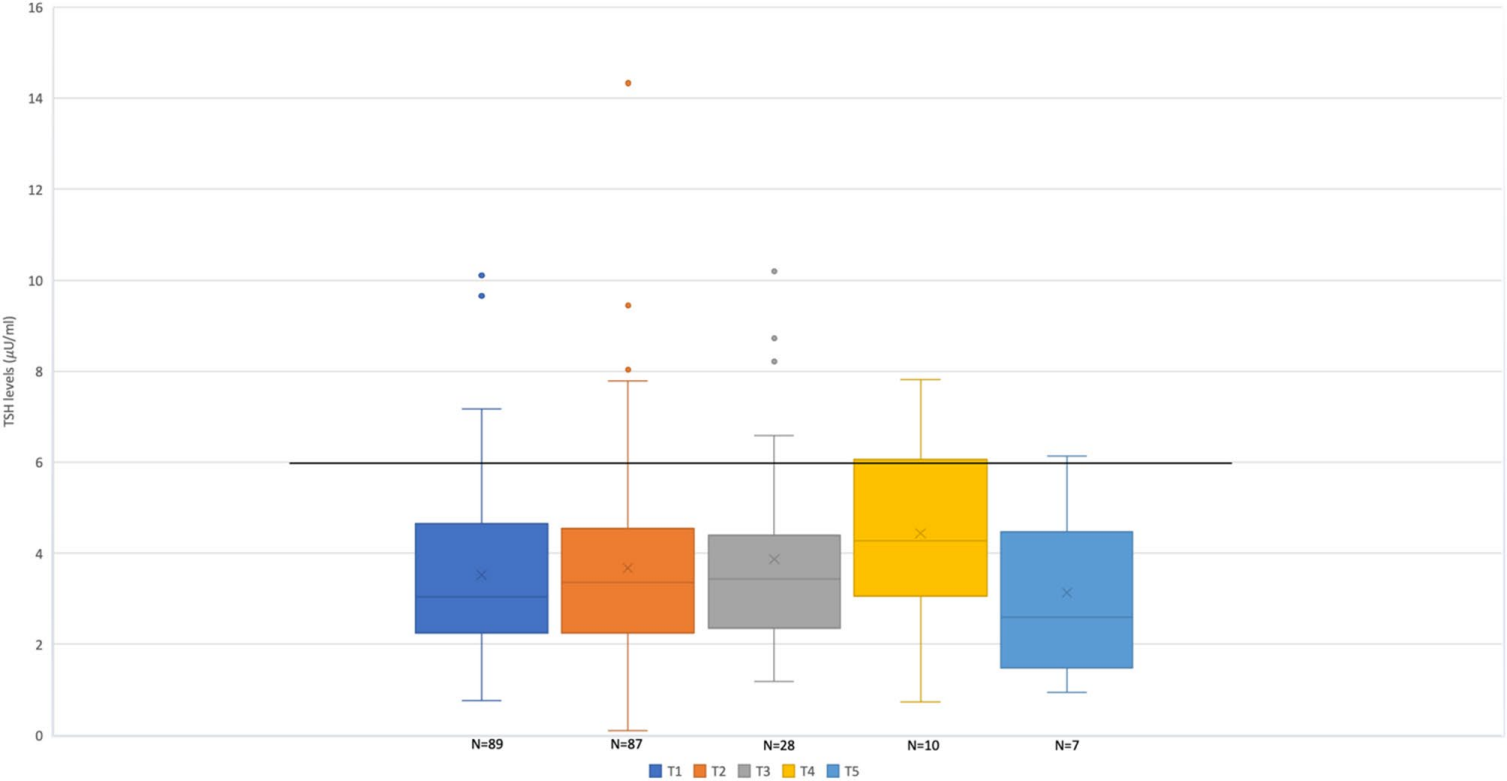
TSH serum levels over time. Data are expressed as median values and quartiles; the cross inside each box indicates the mean. The cut-off line (6 mU/L) is evidenced

Analysing the TSH levels of infants that did not normalise their TSH levels within T3 (*N* = 28), peak TSH levels were detected at T4 (peak TSH: 4.4 ± 2.2 mU/L) (Table 3; Fig. 2).

**Table 3:** Thyroid function data of the 89 infants during follow-up

Name	Days of life	*N* of infants evaluated	TSH levels (mU/L) [range]	*N* infants with TSH > 6 mU/L (% of evaluated)
T1	30 ± 7	89	3.5 ± 1.7 [0.8–10.1]	7 (7.8%)
T2	61 ± 9	87	3.7 ± 2.1[0.9–14.3]	7 (8%)
T3	105 ± 49	28	3.9 ± 2.3 [1.2–10.2]	4 (13.8%)
T4	135 ± 31	10	4.4 ± 2.2 [0.7–7.8]	2 (20%)
T5	247 ± 64	7	3.1 ± 1.8 [0.9–6.1]	1 (14%)

*Abbreviations N* number, *TSH* thyroid stimulating hormone

TSH levels were always normal in 75/89 infants (84%) considering a TSH cut-off of 6 mU/L. Eleven (12%) children had a TSH above 6 mU/L during follow-up, that normalized within 154 ± 100 days after birth.

Two children did not complete the protocol. One received replacement therapy at 2 months of life with L-thyroxine because of TSH levels ≥ 10 mU/L in two consecutive samples (TSH 10 and 14 mU/L, respectively) and is currently in follow-up with a normal thyroid US and no mutation in the TSH receptor gene. Furthermore, circulating anti-thyroid antibodies were absent at that time. Current L-thyroxine dose requirement is 1.3 mcg/kg/day.

FT4 levels were always normal in all infants at all time.

Two out of three children born from mothers with positive anti-TG and negative anti-TPO antibodies presented increased TSH levels up to 74 and 71 days of life and TSH levels of 6,66 mU/L and 7 mU/L, respectively. They normalised their TSH levels within 90 and 135 days of life, respectively.

Out of 22 thyroid US, 20 (91%) were normal. The remaining two presented mild thyroid hypoplasia and mild hypo-echogenicity with scarce dyshomogeneity, respectively. The first infant had mild hyper-TSH and anti-TPO positivity at 2 months and normalised at 3 months of age. The second infant presented transient subclinical hypothyroidism (maximum TSH levels were 10 mU/L at 41 days of life) with normalisation at 3 months of life and positive anti-TPO antibodies up to 7 months of age.

## Discussion

This retrospective observational study showed that only approximately one third of the mothers were checked for anti-thyroid antibodies before pregnancy, whereas two thirds were investigated during pregnancy most at the time of delivery for subclinical or overt hypothyroidism.

Almost all newborns at the time of their first evaluation had positive anti-thyroid antibodies, with decreasing titles or normalization after the second month of life. Furthermore, we observed an increase in TSH levels between 4 and 6 months of life, normalizing over time in almost all children; only one infant received replacement therapy for hypothyroidism at the age of 2 months. Thyroid ultrasound, performed in case of persistent subclinical hypothyroidism, showed minimal changes only in two cases in whom thyroid function normalized over time.

82% of mothers in this study received L-thyroxine therapy during pregnancy, a higher percentage compared to another Italian study (74.2%) [ 16], and lower compared to a Spanish study (96%) [19]. The impact of maternal therapy on the newborns’ thyroid function is not well defined. The American Thyroid Association (ATA) guidelines suggest to assess TSH in pregnant women at high risk as soon as pregnancy is confirmed and add anti-TPO antibodies to the screening if subclinical hypothyroidism (TSH 2.5–10 mU/L) is found; treatment with L-thyroxine is administered whenever TSH is above 10 mU/L or if subclinical hypothyroidism is associated with positive anti-TPO antibodies [1]. In our population, only 62% of the mothers were checked for anti-TPO and/or anti-TG antibodies during pregnancy and later compared to other studies that screened mainly before or during the first trimester of pregnancy [19].

We detected circulating antibodies in almost all newborns, in agreement with previous published data [20]. This is probably due to anti-TPO and anti-TG class IgG antibodies freely crossing the placental barrier from mid gestation, although this passage decreases as pregnancy progresses because of the lowering of maternal-foetal transfer owe to suppression of thyroid autoimmunity over time [2, 21]. Whereas the transplacental passage of TRAb can have a great impact on foetal and neonatal thyroid function, many studies have reported that the passage of anti-TPO and anti-TG antibodies does not seem to significantly influence foetal or neonatal thyroid function [2, 21]. Our results seem to confirm these findings since the only infant treated with L-thyroxine for hypothyroidism was one of the 6% who never presented anti-TPO and anti-TG antibody positivity during the follow-up, and thus the hypothyroidism must be ascribed to other causes.

Interestingly, two of the three children born from the mothers with positive anti-TG antibodies presented a TSH above 6 mIU/L during the third month of life with a subsequent normalisation. An association between elevated anti-TG and TCH has been previously considered [15], however anti-TG antibodies are not actually recommended to be routinely assessed in in pregnant women when tested for thyroid autoimmunity [1]. Our finding would suggest that these antibodies could have an effect on thyroid function, however, further studies are warranted on larger sample sizes to confirm this finding.

In the infants, anti-thyroid antibodies showed a trend to disappear within the 6^h^ month of life in agreement with the literature [19, 22].

Concerning the TSH findings in offspring, our data are similar to previous reported data. Rovelli et al. reported a transient mild elevation of TSH during the first 6 months of life in 28% of the infants born to mothers with AT, and the need for replacement therapy in 2.2% of these [ 22]. The higher percentage of children with normal thyroid function in our study could be due to the different cut-off used for TSH (6 mIU/L in our study, a cut-off stratified for ages according to Ranke’s reference ranges [23] in Rovelli’s study). More recently, Cavarzere et al., reported a slightly elevated serum TSH value (> 6 mU/L) at 15 days of life in 11.4% of infants born to mothers with AT that normalized within 4 months of life and also in his series, one patient with negative neonatal screening required subsequently replacement therapy [16].

Some studies have raised concerns on thyroid dysfunction in infants born to mothers with AT and the need for a specific follow-up of these infants [21].

Some reports have supported thyroid function testing (TFT) during the first weeks of life in newborns from mothers with hypothyroidism, which is usually due to AT [16, 22, 24–26]. One study suggested to reassess thyroid function at 10–15 days of life in these neonates [24], however, it has been hypothesized since that this strategy could over-investigate neonates with transient hypothyroidism [27]. Rovelli et al. recommended follow-up of newborns from mothers with AT measuring TSH levels between the 2nd and 4th week of life [22], similarly a Spanish study, suggested to repeat serum TSH at the same timepoint as above, if serum TSH levels were greater than 6 µU/mL at 48 h of life [25]. Underland et al., analysing thyroid function in 352 newborns from mothers with a history of thyroid disease, suggested to have TFT, in addition to neonatal screening, at 5–10 days of life [26]. More recently, Cavarzere et al. recommended the importance of a second reassessment of thyroid function by blood spot at 15 days of life [16]. At variance with these studies, others authors instead do not support the reassessment of thyroid function in infants born to mothers with hypothyroidism [2, 19, 21, 27–32]. In detail, both McGovern et al. and Haim et al. did not identify any cases of congenital hypothyroidism from the additional testing they performed at 10–14 days of life in infants born to mothers with hypothyroidism [27, 28]. Ben-Zeev et al. supported this hypothesis also, identifying only 3 hypothyroid neonates on a total of 496 newborns born to mothers with thyroid dysfunction [29]. Furthermore, Fernandez Rodriguez et al., studying a group of 72 mother-infant dyads, did not find an effect of mother’s anti-TPO antibodies on infant’s thyroid function nor any cases of transient hypothyroidism in offspring, suggesting a less aggressive follow-up in these children [19]. Urueña, studying 191 neonates mostly born from mothers with AT, identified only 5.8% of newborns with slight increased TSH, normalizing anyway within one month of life and not correlating with anti-TPO levels [30]. Perez et al. studied 790 mothers with hypothyroidism and analysed thyroid function in their offspring at 2 weeks of life, reporting that only 1% of these children presented a TSH > 10 mU/L, which normalised by 4 weeks of age. Moreover, he described that no infant born to women with known AT had a TSH levels above 10 mU/L and suggested that routine thyroid function assessment in addition to NS could offer little additional benefit [21]. Other studies from Ireland and Greece [31, 32] as well as a recent mini-review did not support additional thyroid function testing beyond neonatal screening in identifying undiagnosed cases of CH in children born from mothers with hypothyroidism [33]. Our findings are in line with these studies: in fact, the monitoring of infants born from mothers with AT did not identify new cases of hypothyroidism other than those already identified by NS, since the only child that presented overt hypothyroidism over time never had anti-thyroid positivity during follow-up.

Whereas the majority of studies mentioned above focused primarily on thyroid function during the first weeks of life, our interval of collection of samples was broader: the highest TSH levels (> 6 mU/L), in agreement with the most recent consensus guidelines [18, 34, 35], were registered between 4 and 6 months of life, and normalized in almost all infants by 9 months of age.

Since the majority of patients with a TSH elevation showed normalization at subsequent controls and given the small percentage of children requiring therapy for persistent hyperthyreotropinemia, the literature does not recommend further investigations [1, 16, 22]; in particular, previous studies suggest that thyroid US should be considered only in case of persistent hyperthyrotropinemia [22]. Our findings of a normal thyroid US in 91% of the investigated children is in agreement with the observations above, considering also that the two patients that presented mild thyroid abnormalities at US had a transient TSH elevation and anti-TPO antibody positivity, and none required therapy. However, the difficulty in performing thyroid US in infants, and being done by different healthcare professionals could have biased the results; this strengthens the importance of having expert paediatric radiologists, in order to obtain a correct interpretation of imaging.

The main limitation of this study is its retrospective nature, and the lack of a control group, as well as the small size of the sample. Moreover, data were collected from computerized medical records, therefore, some data present in paper medical records may have been neglected, and the protocol of collection sample was not applied strictly, so that some information from both mothers and infants could be incomplete.

## Conclusion

In conclusion, we report a transient elevation of TSH between 4 and 6 months of life in children born from mothers with AT, and no cases of overt thyroid dysfunction. Since the results of studies currently available on the topic are difficult to compare because of the different approaches to screening and follow-up of infants born from mother with AT, further studies with larger populations are needed. We stress the importance of monitoring thyroid autoimmunity during pregnancy besides thyroid function, especially in all pregnant women requiring L-thyroxine replacement, and having any sign of thyroid dysfunction according to the most recent guidelines [1].

## Data Availability

The datasets used and analysed in the current study are available from the corresponding author on reasonable request.

## Abbreviations

Anti-TPO: Anti-Thyroid Peroxidase antibodies
TSH: Thyroid Stimulating Hormone
AT: Autoimmune Thyroiditis
NICU: Neonatal Intensive Care Unit
IUGR: Intrauterine Growth Restriction
SGA: Small For Gestational Age
IQ: Intelligence Quotient
Anti-TG: Anti-Thyroglobulin antibodies
TCH: Transient Congenital Hypothyroidism
TRAb: Anti-TSH Receptor Antibodies
NS: Neonatal Screening
SD: Standard Deviation
PFO: Gestational age, Patent Foramen Ovale
FT4: Free Thyroxine
FT3: Free Triiodothyronine
US: Thyroid Ultrasound
ATA: American Thyroid Association
TFT: Thyroid Function Tests
